# Extracellular Vesicles: Emergent and Multiple Sources in Wound Healing Treatment

**DOI:** 10.3390/ijms242115709

**Published:** 2023-10-28

**Authors:** Alessandro Sarcinella, Saveria Femminò, Maria Felice Brizzi

**Affiliations:** Department of Medical Sciences, University of Turin, 10126 Turin, Italy; alessandro.sarcinella@unito.it (A.S.); saveria.femmino@unito.it (S.F.)

**Keywords:** EVs, wound healing, therapeutics

## Abstract

Non-healing wound- and tissue-injury are commonly experienced worldwide by the aging population. The persistence of disease commonly leads to tissue infection, resulting in severe clinical complications. In the last decade, extracellular vesicles (EVs) have been considered promising and emergent therapeutic tools to improve the healing processes. Therefore, efforts have been directed to develop a cell-free therapeutic platform based on EV administration to orchestrate tissue repair. EVs derived from different cell types, including fibroblast, epithelial, and immune cells are recruited to the injured sites and in turn take part in scar formation. EVs are nano-sized particles containing a heterogeneous cargo consisting of lipids, proteins, and nucleic acids protected from degradation by their lipid bilayer. Noteworthy, since EVs have natural biocompatibility and low immunogenicity, they represent the ideal therapeutic candidates for regenerative purposes. Indeed, EVs are released by several cell types, and even if they possess unique biological properties, their functional capability can be further improved by engineering their content and functionalizing their surface, allowing a specific cell cargo delivery. Herein, we provide an overview of preclinical data supporting the contribution of EVs in the repair and regenerative processes, focusing on different naïve EV sources, as well as on their engineering, to offer a scalable and low-cost therapeutic option for tissue repair.

## 1. Introduction

Tissue repair is a complex, dynamic, and highly regulated process that involves the activation of specific signaling pathways promoting the remodeling of the tissue microenvironment composed of various cell types. The first event, defined as hemostasis, consists of the activation of the intrinsic and extrinsic coagulation pathways to prevent blood loss; then immune cells, in particular monocytes, are recruited to the injury site where they remove debris and bacteria. The recruitment of inflammatory cells and platelets promotes fibroblast migration, which contributes to extracellular matrix (ECM) release thereby resulting in scarring [1]. This process is considered a universal event occurring across all multicellular organisms, thereby it can be speculated that most of the conserved mechanisms can be investigated in experimental models and inferred to the clinic for human application. A significant understanding of the straightforward cellular and molecular mechanisms of regeneration has been gained by studying different preclinical models [2]. Recently, a large number of studies have highlighted the potential of extracellular vesicles (EVs) in enhancing tissue remodeling [3,4]. Briefly, EVs are a heterogenous group of bilayer particles, which are classified based on different physical characteristics, including their size. Therefore, the updated classification refers to EVs as small/medium and large EVs [5]. Once released, EVs can reach recipient cells both locally and at distant sites and deliver their contents to elicit functional responses and promote phenotypic changes. All these changes are mainly dependent on the physiological or pathological status features of their cell of origin. This implies that the interactions with and the transfer of EVs to recipient cells can drive various biological effects on the target cell, ranging from the activation of specific physiological activities to the development of pathological programs [6]. Currently, research dissecting the mechanisms by which EVs from different sources display certain peculiarities impacting the healing processes is ongoing. The structural similarity of EVs to liposomes makes them ideal for therapeutic delivery. Additionally, EVs are considered a more advantageous platform over liposomes since they can better reproduce the cell membrane [7]. In addition, recent studies showed promising applications of EVs due to their cargo specificity and natural ability to cross various biological barriers, preserving and protecting their content from degradation [4,8]. During EV-based therapeutic development, the major issue is represented by the selection of the best cellular source [9]. For example, EVs from inflammatory cells [10] mediate different biological actions with respect to EVs from stem cells [11]. EV-based compounds are administered mostly by subcutaneous injection to multiple sites surrounding the wound area, showing a fascinating boost in wound healing and tissue regeneration via a variety of biological processes, including revascularization, neo-angiogenesis, cell proliferation, and motility [3]. Recently a pilot case-control study in humans showed the efficacy of serum-derived EVs in the treatment of ulcers not-responsive to conventional medications [12]. However, to move towards clinical application using EVs derived from cellular sources, particular attention must be devoted to cell culture conditions, yield, and scalability [13]. Therefore, in the last decade, several research articles focused their attention to the manufacture of EVs also derived from different types of fruit or vegetables [14], paving the way toward a new era of wound healing treatment, exploiting the potential of plant-derived EVs. Moreover, EVs can be further engineered by manipulating parental cells to produce particles with the desired features or can be directly modified to act as delivery tools for specific therapeutic agents [15]. All these features drive EV-based therapy toward a more personalized clinical application. Here, we cover innovative approaches and recent advances in EV-based therapeutics in wound healing by summarizing the current knowledge of novel potential treatments.

## 2. Stem-Cell-Derived EVs

Recently, stem-cell-based therapy has gained attention due to its pluripotency and the potent ability to secrete a large number of regenerative factors [16]. Indeed, stem cells, including embryonic stem cells (ESC), induced pluripotent stem (iPS) cells, and adult stem cells, demonstrated unique properties such as a high proliferation rate and boosted self-renewal potentials compared to committed and specialized cells [17]. Indeed, stem cells have been proposed to promote wound healing in a paracrine way, regulating different cell subsets, and EVs represent one of the most relevant paracrine mediators and the main contributors to stem cell efficacy [18]. In particular, stem-cell-derived EVs have been proven to stimulate angiogenesis and cellular regeneration by suppressing apoptotic pathways and modulating immunity in damaged tissues both in vitro and in vivo [19]. Noteworthy, the clinical application of stem-cell-based therapy has faced problems with storage, allocation, and risks of tumorigenesis [20]. Therefore, EVs, which, in contrast to stem cells, cannot self-replicate, are much more feasible in terms of potential tumour formation and, more importantly, are stable enough for long-term storage, even at room temperature after lyophilization [21]. In addition, EVs can be administered by several routes; for example, nebulized or lyophilized lung-stem-cell-derived EVs can be administered by inhalation to treat lung diseases [21,22]. All these properties provide the rationale to exploit stem-cell-derived EVs as a valuable alternative approach to stem cells. In this regard, EVs from human umbilical cord mesenchymal stem cells (hucMSCs) promote angiogenesis and wound healing by activating the Wnt/β-catenin pathway by exosomal Wnt4a in a rat skin burn model [23], and reduce scar formation and myofibroblast accumulation in a skin-defect mouse model [24] by inhibiting the TGFβ2/SMAD2 pathway through exosomal microRNAs (miR-21, -23a, -125b, and -145). Furthermore, EVs isolated from human-induced-pluripotent-stem-cell-derived mesenchymal stem cells (hiPSC-MSCs) accelerate the re-epithelialization process through collagen promotion by triggering type I and III collagen and elastin secretion and mRNA expression by fibroblasts, resulting in scar-size decrease in a cutaneous-wound rat model [25]. A similar study demonstrated that EVs from human-induced-pluripotent-stem-cell-derived keratinocytes (hiPSCs-KCs) boost skin repair in a murine model of deep second-degree burn thanks to the enrichment of miR-762, which is involved in the promotion of keratinocyte and endothelial cell migration [26]. Moreover, in an aged mouse model of pressure-induced ulcer, EVs purified from human embryonic stem cells (ESC) were able to rejuvenate senescent endothelial cells and recover their proliferation and migratory capacity by Nrf2 activation via transference of miR-200a [27]. Similarly, Li X. et al. [28] demonstrated the therapeutic effect of EVs secreted by adipose-derived stem cells (ADSCs) overexpressing Nrf2 in the high glucose-induced senescence of endothelial progenitor cells using a diabetic rat model of diabetic foot ulcers. At this regard, ADSCs arouse great interest due to their great availability and easy isolation procedures that make their derivatives, like EVs, more appealing in preclinical and clinical settings [29,30]. Interestingly, Pomatto M. et al. [11] showed different therapeutic effect of EVs from ADSCs and bone marrow-derived stem cells (BMDSCs) in a mouse diabetic model of ulcers. Indeed, they demonstrated that BMSC-EVs mainly affected cell proliferation and viability of fibroblasts, keratinocytes, and endothelial cells, while ADSC-EVs showed a significant improved ability to promote endothelial cell migration and angiogenesis in vitro. However, in the in vivo experiments, only ADSCs-EVs were able to significantly reduce the wound size and induce an increased epithelial thickness, re-epithelization, and neo-vessel formation [11]. All these data indicate the mechanisms trough which stem cell-derived EVs can act at different levels during the wound healing processes. Therefore, since it appears that the cellular stem cell source and the EV cargoes could affect distinct repair processes the importance of selecting the best EV source to optimize the therapeutic efficacy is mandatory.

## 3. Macrophage-Derived EVs

Immune cells evolved as the primary defense against pathogenic wound invasion. Indeed, the immune response is initiated by injury-induced signals released by necrotic cells, damaged tissue, and bacterial components, which in turn activate resident immune cells to elicit downstream inflammatory pathways [31,32]. Specifically, in response to pro-inflammatory signals, neutrophils are recruited to the injured site to remove necrotic tissue and pathogens [33]. However, following the entrance of neutrophils, circulating monocytes move into the wound tissue where, in response to the local milieu, differentiate into macrophages. Thus, macrophages may be considered master effector cells in tissue repair [34]. Wound macrophages are classified into two main subsets: M1 (pro-inflammatory) and M2 (anti-inflammatory), possessing both pro-inflammatory and healing properties. Noteworthy, anti-inflammatory macrophages release a huge amount of growth factors to promote re-epithelialization, fibroplasia, and angiogenesis [35,36]. The effect of EVs derived from macrophages displaying different phenotypes on cutaneous healing has been recently analyzed [37]. Importantly, in a mouse model of skin-wound, studying bone-marrow-derived-macrophages, it was found that M2-macrophage-derived EVs (M2-EVs) induce macrophage reprogramming from the M1 to the M2 phenotype, resulting in sustained angiogenesis, re-epithelialization, and collagen deposition. Similarly, it has been recently demonstrated how macrophage-derived EVs recovered from adipose tissue efficiently modulate macrophage polarization, promoting the shift from M1 to M2, through the miR-222-3p/Bim axis. This translates into boosting diabetic wound healing when administered to diabetes-prone mice [38]. Indeed, it has been recently discovered that M1 polarization predominates in diabetic skin tissue, affecting the healing process; in particular, M1-derived EVs containing miR-503 lead to endothelial cell dysfunction, targeting IGF1R and thus inhibiting cell viability, tube formation, and cell migration [10]. Based on these notions, it can be speculated that the phenotype switch induced by EVs harboring a farfetched cell-reprogramming capability can represent a promising therapeutic approach for wound healing purposes by regulating the balance between the M1–M2 state. However, macrophage-derived EVs can exert their effect not only by reverting the phenotype but also by directly targeting cells within the wound site. Li M. et al. have found that EVs derived from RAW 264.7 cells significantly attenuated the secretion of pro-inflammatory cytokines and promoted the proliferation and migration of endothelial cells, improving de novo vessel formation in a skin-defect diabetic rat model [39]. Interestingly, in a recent work by Deng F. et al., M2-polarized Thp-1 cells secreted EVs enriched in miR-590-3p which promoted colonic epithelial cell proliferation via the LATS1/YAP/β-catenin signaling axis in a model of DSS-induced mucosal damage [40]. Notably, it has been recently reported that EVs isolated from the RAW 264.7 mouse macrophage cell line contain more pro-angiogenic factors, including VEGF, Wnt3a, and miR-130a, when compared to endothelial cells (HUVECs). Furthermore, it was observed that RAW-264.7-cell-derived EVs used for in vitro treatment significantly increase endothelial cell proliferation, migration, and tube formation, while use in vivo increases the formation of new and larger blood vessels [41]. These observations remark the pivotal role of the immune system, particularly macrophages, on the resolution of wound damage.

## 4. Platelet-Derived EVs (P-EVs)

Since P-EVs have an impact on several pathophysiological processes, such as angiogenesis, coagulation, and inflammation, they have been recently recognized as a promising therapeutic strategy for tissue regeneration [42]. Although there are many obstacles to using P-EVs in clinical practice, they are gaining attention due to their efficiency compared to platelets [43]. Indeed, in a model of immortalized human corneal endothelial cells, treatment with P-EVs did not exert cellular toxicity, maintaining cellular morphology and the preservation of corneal proteins. Corneal cells, which have a very limited regenerative ability, treated with P-EVs have shown increased proliferation and migration in a dose-dependent manner [43]. P-EV treatment has been also proposed for periodontal applications. Both gingival fibroblasts and keratinocytes were treated with P-EVs or with P-EVs combined with hyaluronic acid gels [44]; compared to the direct use of platelets, these treatments have shown regenerative effects and changes in the expression of genes related to extracellular matrix remodeling, including *ACTA2, COL1A1* and *DCN* [44]. Guo et al. [45] reported that P-EVs increase proliferation and migration of endothelial cells (HMEC-1) and fibroblasts through activation of Erk and Akt signaling pathways. Consistently, in a clinical trial, EVs purified from platelets of healthy donors, determined the proliferation and migration of dermal fibroblasts compared to untreated cells through the phosphorylation of Erk and Akt [46]. In order to obtain a sustained drug release, P-EVs were combined with hydrogels and Resveratrol, an anti-inflammatory agent [47]. This combination, in a skin-wound model of diabetic mice, inhibits the inflammatory response and regulates macrophage phenotype transition and angiogenesis, leading to wound healing improvements [47]. Another in vivo wound healing study reported that chitosan/silk hydrogels loaded with P-EVs accelerate skin regeneration in diabetic rats [48]. In particular, accelerated wound healing, re-epithelialization, increased collagen synthesis, as well as dermal angiogenesis are described, thereby resulting in faster diabetic wound healing [48]. In conclusion, P-EVs alone or combined with different types of gels could be a promising bioengineering strategy in regenerative biotherapies.

## 5. Endothelial-Cell-Derived EVs (E-EVs)

Angiogenesis is a crucial step during the process of tissue regeneration. During wound healing, pre-existing vessels invade the wound clot and, within a few days, organize into a microvascular network. The cooperative regulation of endothelial cells, angiogenic cytokines, and the extracellular matrix environment stimulates angiogenesis to promote wound repair [49]. It has been extensively demonstrated that the stimulation of resident endothelial cells via paracrine mechanisms is more relevant than the direct differentiation into mature endothelial cells, and EVs play a major role in such paracrine action [50,51]. Indeed, it has been reported that EVs derived from endothelial progenitor cells (EPCs) were purified from healthy newborns and used to treat diabetic rats [52]. A wound-healing assay revealed that E-EVs affect the migration of endothelial cells and modulate the expression of various angiogenesis-related genes, including *endothelial fibroblast growth factor*1, *interleukin*-8, *nitric oxide synthase*, *angiopoietin*-1, *E-selectin*, *vascular endothelial growth factor A*, *vascular endothelial growth factor receptor* 2, and the *chemokine ligand*-16, thereby promoting angiogenesis [52]. Taken together, these findings draw attention to the role of E-EVs in promoting wound healing by modulating vascular endothelial cell behavior, thereby suggesting them as therapeutic tools for diabetic patients. In the context of diabetes, foot ulcers represent a serious clinical problem. Zeng et al. [53] demonstrated that miR-106b-5p could be a novel target to improve the healing of diabetic foot ulcers. Indeed, miR-106b-5p-containing small E-EVs were able to inhibit collagen synthesis by activating autophagy of human skin fibroblasts. This effect relies on Erk1/2-targeting in vitro and translates into a wound healing delay in vivo. Therefore, E-EVs enriched in miR-106b-5p would be a promising carrier to deliver siRNA or therapeutic drugs to promote wound healing in diabetic patients with foot ulcers. Consistently, miR-106b has been found as an inhibitor of skin-wound healing, thereby becoming a new therapeutic target [54].

## 6. Engineered EVs

In the last decade, new engineering technologies have been developed to improve EV therapeutic action [55]. Different methodologies are actively being explored to improve EV therapeutic actions in wound healing processes. Indirect engineering strategies include cell modifications, while direct techniques are applied directly to EVs [15]. In this specific context, the enrichment of miR-21 in EVs obtained from ADSCs (indirect approach) was found to improve the migration and proliferation of immortalized human keratinocytes (HaCaT cells) by modulating MMP-9 and MMP-2 expression through the PI3K/AKT signaling pathway [56]. Engineered EVs (direct approach) with miR-31-5p stimulate wound healing both in vitro and in vivo, by enhancing angiogenesis, re-epithelization, and fibrogenesis. Down-regulation of the factor-inhibiting HIF-1 (HIF1AN) and the epithelial membrane protein-1 (EMP-1) has been proposed due to their mechanism of action [57]. In another study, miR-155-inhibitor loaded in EVs derived from MSCs (direct approach) was reported to promote re-epithelization and neovascularization in vitro by regulating FGF and MMP expression. In addition, the authors demonstrated the improvement of diabetic wound healing in vivo by down-regulating the levels of inflammatory cytokines [58]. A different study has demonstrated that MSCs transfected with HOX transcript antisense lncRNA (HOTAIR) (indirect approach) were able to release EVs enriched in HOTAIR, acquiring the ability to ameliorate wound healing in diabetic mice [59]. Transfected EVs induced neovascularization in a diabetic mouse (db/db) model, while native MSC-EVs did not have the same biological effect. Small molecules, such as melatonin, were loaded in EVs (direct approach). Indeed, Liu et al. [60] reported that MSC-derived EVs loaded with melatonin promote macrophage polarization from the M1 to the M2 phenotype and the expression of anti-inflammatory factors, such as IL-10 and ARG, through the activation of the PTEN/Akt signaling pathway; this enhances wound healing in a diabetic rat model. All these studies emphasize the importance of EV genetic manipulations to optimize the therapeutic efficacy of EV-based approaches in the resolution of the wound in different pathological contexts accomplished without relevant side effects.

## 7. Plant-Derived EVs

During the past few years, plant-derived EVs have gained particular interest as a promising therapeutic strategy in clinical applications. Surprisingly, it has been shown that EVs isolated from plant cells have very similar characteristics and secretion patterns to mammalian cells [61,62]. In particular, plant-derived EVs represent one of the more advantageous systems to deliver substances in comparison to existing analogues. Indeed, the small size and negative charge, coupled with the enhanced physicochemical stability at different ranges of pH and temperature, allow for an efficient penetration of target cells. Furthermore, these types of EVs have a high potential for targeted delivery with a low probability of adverse reactions when compared to artificially-made liposomes and animal-derived EVs [63]. Their therapeutic effects have been demonstrated in different human diseases, including wound healing and tissue repair [62]; this mainly relies on their powerful antioxidants and anti-inflammatory effect [64]. In this regard, it was found that grapefruit-derived extracellular vesicles (GEVs) decrease the intracellular ROS content via H_2_O_2_-driven oxidative stress in the human epidermal keratinocyte cell line (HaCaT cells). Indeed, GEVs, by means of their antioxidant properties, promoted HaCaT cell proliferation and migration and increased the expression of wound-healing-related mRNAs and proteins, including collagen type I, fibronectin, laminin, and vimentin. Furthermore, a tube-formation assay confirmed that GEVs also promote the formation of capillary tubes, which improve wound healing by supplying oxygen and nutrients [65]. Similarly, Sánchez-López et al. [66] first isolated EVs from pomegranate juice (PgEVs), demonstrating their anti-inflammatory effects on Thp-1 cells and the ability to improve the healing process in a scratch assay using Caco-2 cells. Moreover, when EVs from Aloe Saponaria (ASEVs) were used to treat RAW 264.7, HDFs, or HUVEC with the aim to investigate chronic skin-wound healing, no toxic effects were detected. Notably, ASEVs effectively reduced the mRNA expression of inflammatory cytokines, such as IL-6 and IL-1β, resulting from lipopolysaccharide (LPS) stimulation of the RAW 264.7 mouse macrophage cell line. Moreover, these ASEV-mediated anti-inflammatory effects translate into an increase in proliferation and migration of HDF cells [67]. In a different study from Kim et al. [68], it was shown that the wound-healing properties of EVs derived from Aloe vera (AEVs) were also associated with a marked antioxidant effect. The antioxidant activity of AEVs was found to increase in a dose-dependent manner and via AEV internalization. The authors also demonstrated that AEVs (10^9^ particles/mL) exhibited antioxidant properties similar to those of quercetin, a flavonoid with potent antioxidant effects. Finally, AEVs upregulated the mRNA expression of genes involved in antioxidant defense, such as Nrf-2. All these effects boosted the migration of both HaCaT cells and human dermal fibroblasts (HDFs).

Noteworthy, wheat-derived EVs were also found to display regenerative properties. This effect relies on their proliferative and migratory action on endothelial cells, epithelial cells, and dermal fibroblasts, and depends on the increased expression level of collagen type I mRNA, the formation of tube-like structures, and the decrease of apoptosis [69]. Overall, plant-derived EVs may represent a novel approach for tissue remodeling and future clinical application.

## 8. Erythrocyte-Derived EVs (RBC-EVs)

Recently, EVs derived from erythrocytes (RBCs) have attracted attention for different clinical applications. RBC-EVs are released into circulation and interact with different cells under both physiological and pathological conditions [70]. Originally, it was shown that RBC-EVs can act to remove hemoglobin or other RBC proteins, thereby preserving RBC function [71]. In addition, RBC-EVs were reported to act as key regulators of redox balance, nitric oxide homeostasis, and immunomodulation [72]. In the last decade, particular interest has been dedicated to investigating RBC-EVs as drug delivery systems, while only a few studies have been performed to explore their regenerative properties [73]. Recently, Shao et al. [74] demonstrated that RBC-derived apoptotic vesicles promote bone regeneration, by delivering carbonic anhydrase 1 into bone mesenchymal stem cells, and can drive their osteogenic differentiation. This relevant data sustains the need for a deeper understanding of RBC-EV biological properties, which, along with their well-known potential as a tool for drug delivery, may provide the rationale to move towards their widespread application in clinics. The most relevant effect of EVs in wound healing are reported in Table 1.

## 9. Conclusions

Several pieces of evidence related to both cellular and molecular physiological healing processes have been reported, however, hurdles and limitations remain to be overcome to guide clinicians towards better prognostic indicators and better therapeutic approaches for wound healing. Intense research on EVs over the last decade has increased our understanding of their biogenesis, molecular content, and biological function. Indeed, EVs, thanks to their biocompatibility, display a high potential as a drug-delivery system. In this regard, EVs are emerging as highly potent therapeutic entities due to their innate properties, as well as the possibility of their loading with small therapeutic molecules, nucleic acids, and proteins. A large number of loading procedures and isolation methods are currently being developed and optimized for EV isolation, but identifying an optimal methodology allowing scalability of pure and clinical-grade EVs is still a challenge. Therefore, clinical application of EVs as off-the-shelf therapeutics and their stability and storage must be further improved. In addition, to move towards their clinical application, standardized potency assays able to predict their in vivo efficacy is still an unmet need. Overall, the aim of this review was to better describe the fundamental molecular mechanisms by which EVs derived from different sources and carrying different cargoes can contribute to the wound healing process. However, although all sources of EVs have been reported to trigger fundament biological processes, such as angiogenesis, re-epithelization, and anti-inflammatory responses, several issues should be addressed before identifying the best sources exploitable in humans.

## Figures and Tables

**Table 1:** Summary of studies reporting EV-mediated effects in wound healing.

EV Origin	Contents/Mediators	Effects	Refs.
M1	miR-503; IGFR-1	Angiogenesis; cell migration	[10]
ADSCs	TGF-β; HIF-1α	Re-epithelization; angiogenesis	[11]
hucMSCs	Wnt4a	Angiogenesis	[23]
hucMSCs	miR-21; -23a; -125b; -145	Myofibroblast differentiation	[24]
hiPSC-MSCs	OCN; Sox9; LPL	Collagen synthesis; angiogenesis	[25]
iPSCs-KCs	miR-762	Keratinocytes and endothelial cells migration	[26]
ESCs	miR-200a	Endothelial cells rejuvenation	[27]
ADSCs	Nrf2	Endothelial cells rejuvenation	[28]
M2	miR-222-3p/Bim	M1 to M2 shift; angiogenesis; collagen deposition	[38]
RAW 264.7 cells	TNF-α; IL-6; Akt; VEGF	Angiogenesis; anti-inflammatory responses	[39]
THP-1	miR-590-3p; LATS1/YAP/β-catenin	Re-epithelization	[40]
RAW 264.7 cells	VEGF; Wnt3a; miR-130a	Endothelial cell proliferation and migration; angiogenesis	[41]
Platelets	PDGF; FGF-2; TGF; VEGF	Corneal cells proliferation and migration	[43]
Platelets	ACTA2; COL1A1; DCN	Periodontal regeneration	[44]
Platelets	Erk; Akt; Rho/YAP	Re-epithelization; angiogenesis	[45]
Platelets	Erk; Akt	Dermal fibroblasts proliferation and migration	[46]
Platelets	TNF-α; iNOS; TGF-β1; Arg-1	M1 to M2 shift; angiogenesis; anti-inflammatory responses	[47]
Platelets	n/a	Re-epithelialization; increased collagen synthesis; dermal angiogenesis	[48]
EPCs	FGF-1; VEGFA; ANG-1; E-selectin; CXCL-16; eNOS; Il-8	Angiogenesis	[52]
Endothelial cells	miR-106b-5p; Erk	Autophagy of human skin fibroblasts	[53]
Endothelial cells	miR-106b-5p; JMJD3; RIPK3	Delay of skin wound healing	[54]
ADSCs	MMP-9; PI3K/Akt	Migration and proliferation of HaCaT cells	[56]
HEK293 cells	HIF1AN; EMP-1	Angiogenesis; re-epithelization; fibrogenesis	[57]
MSCs	FGF; MMP	Re-epithelization; angiogenesis	[58]
MSCs	n/a	Angiogenesis	[59]
MSCs	PTEN/Akt	M1 to M2 shift; anti-inflammatory responses	[60]
Grapefruit	n/a	Anti-oxidant effect; angiogenesis	[65]
Pomegranate	n/a	Anti-inflammatory responses	[66]
*Aloe saponaria*	IL-6; IL-1β	Anti-inflammatory responses; fibroblast migration	[67]
*Aloe vera*	Nrf2	Anti-oxidant effect	[68]
Wheat	n/a	Anti-apoptotic effect; angiogenesis	[69]
RBCs	CA1	Osteogenic differentiation	[74]

n/a= not applicable.
